# The effects of aging on host resistance and disease tolerance to SARS‐CoV‐2 infection

**DOI:** 10.1111/febs.15613

**Published:** 2020-11-17

**Authors:** Darren Z. L. Mok, Candice Yuen Yue Chan, Eng Eong Ooi, Kuan Rong Chan

**Affiliations:** ^1^ Emerging Infectious Diseases Program Duke‐NUS Medical School Singapore Singapore; ^2^ Department of Infectious Diseases Singapore General Hospital Singapore; ^3^ Viral Research & Experimental Medicine Center @ SingHealth/Duke‐NUS (ViREMiCS) Singapore Singapore; ^4^ Singapore‐MIT Alliance in Research and Technology Antimicrobial Resistance Interdisciplinary Research Group Singapore Singapore; ^5^ Saw Swee Hock School of Public Health National University of Singapore Singapore; ^6^ Department of Microbiology and Immunology Yong Loo Lin School of Medicine National University of Singapore Singapore

**Keywords:** aging, COVID‐19, ER stress, immunometabolism, immunosenescence, resistance, SARS‐CoV‐2, tolerance

## Abstract

The ongoing coronavirus disease 2019 (COVID‐19) crisis caused by severe acute respiratory syndrome coronavirus 2 (SARS‐CoV‐2) has triggered a large‐scale pandemic that is afflicting millions of individuals in over 200 countries. The clinical spectrum caused by SARS‐CoV‐2 infections can range from asymptomatic infection to mild undifferentiated febrile illness to severe respiratory disease with multiple complications. Elderly patients (aged 60 and above) with comorbidities such as cardiovascular diseases and diabetes mellitus appear to be at highest risk of a severe disease outcome. To protect against pulmonary immunopathology caused by SARS‐CoV‐2 infection, the host primarily depends on two distinct defense strategies: resistance and disease tolerance. Resistance is the ability of the host to suppress and eliminate incoming viruses. By contrast, disease tolerance refers to host responses that promote host health regardless of their impact on viral replication. Disruption of either resistance or disease tolerance mechanisms or both could underpin predisposition to elevated risk of severe disease during viral infection. Aging can disrupt host resistance and disease tolerance by compromising immune functions, weakening of the unfolded protein response, progressive mitochondrial dysfunction, and altering metabolic processes. A comprehensive understanding of the molecular mechanisms underlying declining host defense in elderly individuals could thus pave the way to provide new opportunities and approaches for the treatment of severe COVID‐19.

## Graphical abstract

Elderly patients infected with SARS‐CoV‐2 are at higher risk of developing severe COVID‐19 compared with younger individuals. This outcome could be due to aging‐related alterations in host resistance and disease tolerance mechanisms against pulmonary immunopathology. We review the current knowledge on how aging affects these mechanisms and advocate for more detailed studies on how aging affects viral infection to improve the management of its associated clinical outcome.

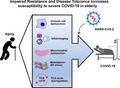

**Abbreviations**
ACE‐2: Angiotensin‐converting enzyme‐2
AMPK: 5’ AMP‐activated protein kinase
ANG: Angiotensin
ARDS: Acute respiratory distress syndrome
ATP: Adenosine triphosphate
COVID‐19: Coronavirus disease‐19
CRP: C‐reactive protein
DAMPs: Danger‐associated molecular patterns
ER: Endoplasmic reticulum
GDP: Gross domestic product
HCV: Hepatitis C virus
IDO: Indoleamine 2,3‐dioxygenase
LDH: Lactate dehydrogenase
MERS‐CoV: Middle East respiratory syndrome coronavirus
NSAIDs: Nonsteroidal anti‐inflammatory drugs
PAMPs: Pattern‐associated molecular patterns
PRRs: Pattern recognition receptors
ROS: Reactive oxygen species
RT–PCR: Reverse transcription polymerase chain reaction
SARS‐CoV: Severe acute respiratory syndrome coronavirus
SARS‐CoV‐2: Severe acute respiratory syndrome coronavirus‐2
TCA: Tricarboxylic acid cycle
TDO: Tryptophan 2,3‐dioxygenase
UPR: Unfolded protein response
YF‐17D: Yellow fever 17D

## Introduction

The first half of 2020 has seen the world plunged into a crisis by the pandemic spread of a novel coronavirus–severe acute respiratory syndrome coronavirus‐2 (SARS‐CoV‐2). Within a span of 6 months, countries and territories across the globe have reported millions of cases of coronavirus disease‐19 (COVID‐19). Without a preventative vaccine, the prevention of COVID‐19 has relied on physical distancing in order to reduce the risk of SARS‐CoV‐2 transmission. Such measures have disrupted the world economy, with global trade and gross domestic product (GDP) of affected countries expected to fall by up to 30% and 10%, respectively [1]. These global effects have led the World Health Organization to declare COVID‐19 as a public health emergency of international concern on January 30, 2020 [2].

SARS‐CoV‐2 infection results in a spectrum of clinical outcomes. Most infected individuals are either asymptomatic (estimated to range from 17.9 to 78%) or present with mild disease. However, approximately 15% of infected individuals develop severe disease and about 5% eventually develop life‐threatening pneumonia and acute respiratory distress syndrome (ARDS) [3, 4]. Some COVID‐19 patients also develop systemic manifestations such as secondary sepsis, cardiovascular and cardiac complications, thromboembolism, coagulopathy, and multi‐organ failure [4, 5]. A major risk factor for severe COVID‐19 is age. The elderly are also more likely to have underlying comorbidities such as hypertension, cancer, obesity, cardiovascular diseases, and diabetes mellitus, all of which are associated with increased risk of severe COVID‐19 [4, 6, 7, 8, 9]. However, these comorbidities alone are insufficient to account for why age is an independent risk factor. Indeed, multiple reports have highlighted the disparity in clinical outcomes between younger and elderly patients with SARS‐CoV‐2 infection [5, 8, 10, 11, 12]. A cross‐sectional study of residents and staff of nursing homes and assisted living facilities in Massachusetts demonstrated that younger individuals infected with SARS‐CoV‐2 were more likely to be asymptomatic compared with older patients despite similar viral loads between both groups of patients [10]. These patterns of symptomaticity are akin to that of cohort studies conducted in South Korea and Wuhan, China [11, 12]. Interestingly, a systematic characterization of the clinical, molecular, and immunological data from 326 confirmed cases of COVID‐19 in Shanghai showed that age, among other factors, was significantly correlated with poor clinical outcomes [8]. Similar trends have also been reported from infection with the related betacoronaviruses, SARS‐CoV, and Middle East respiratory syndrome CoV (MERS‐CoV). During the 2003 SARS epidemic, mortality was over 50% in those above 65 years of age, while no SARS patients under 24 years of age died [13]. Likewise, the mortality from MERS is about 1.7 times higher in elderly patients above 60 years of age compared with younger patients [14]. Notably, the other respiratory infections including seasonal influenza, tuberculosis, and pneumococcal disease also report that age is a major risk factor for severe disease outcomes [15]. Collectively, these observations provide preliminary evidence to support that the aging phenotype predisposes infected individuals to a more severe disease outcome.

**Fig 1:**
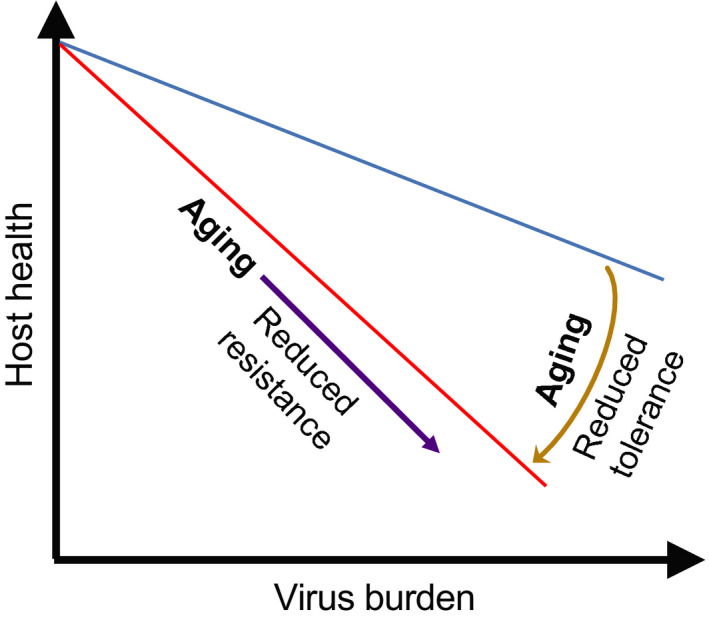
Effects of host tolerance on host health. When the host fitness is plotted against the viral burden, the slope of the line will indicate the host tolerance to the viral infection. In elderly subjects, the reduced resistance (purple arrow) and reduced tolerance (brown arrow) lead to compromised host health upon viral infection. Reduced resistance results in increased susceptibility to viral infections, resulting in increased virus burden that reduces host health. In contrast, reduced tolerance results in a steeper slope (red line), where per unit increase in viral burden will have a greater impact on host fitness in elderly subjects. Interventions that promote host resistance and tolerance can reduce viral burden and the slope of the line respectively, promoting host health.

Fundamentally, two major defense strategies—resistance and disease tolerance—are employed by the host to cope with viral infections. Resistance serves to reduce the viral burden so as to limit the host cell damage caused by the pathogen [16]. Disease tolerance, on the other hand, promotes host health by preventing or resolving immunopathology in host tissues [17, 18]. This should not be confused with immune tolerance, which is defined as a state of nonresponsiveness to self or foreign tissue antigens [19]. In this article, the use of the word ‘tolerance’ refers to ‘disease tolerance’. Both resistance and disease tolerance work in tandem to define the host’s defensive capacity and ability to survive an infection. The contribution of host resistance and disease tolerance to overall health can be generally represented by plotting the health status against the pathogen burden (Fig. 1). However, during aging, the host tolerance can be compromised by several factors, including reduced immune functions, weakening of the unfolded protein response, physiological changes, increased mitochondrial dysfunction, and altered metabolic processes. The reduced host tolerance in elderly individuals thus results in a steeper slope where the host health would have a more dramatic effect per unit increase in viral burden (Fig. 1). Herein, we examine the current literature to glean how the molecular and cellular changes that occur during aging can disrupt resistance and disease tolerance mechanisms. We explore how disruption of such resistance and disease tolerance mechanisms underpin the association between age and severe COVID‐19. Finally, we also suggest potential therapeutic avenues to reduce the risk of severe COVID‐19 in the elderly.

## Aging and the immune system

Aging is known to affect the immune system at multiple levels with dysfunction across both innate immunity and adaptive immunity, which contributes to reduced resistance to infection, a phenomenon known as ‘immunosenescence’. On the other hand, aging is associated with a state of chronic subclinical inflammation, sometimes referred to as ‘inflammaging’, which can compromise disease tolerance during infections [20, 21, 22]. These are likely to be accountable for the increased morbidity and mortality of older individuals due to infections, including that caused by SARS‐CoV‐2 [20, 21, 23, 24]. Furthermore, immunosenescence raises concerns about the feasibility of generating a potent vaccine to induce efficient cellular and humoral immunity in the aged population. Gaining knowledge of these mechanisms and interactions will be critical for the discovery of novel therapeutic interventions, especially for older individuals. Herein, we provide a brief summary of the concepts of immunosenescence and inflammaging, which have been extensively reviewed elsewhere [21, 25, 26, 27, 28, 29, 30, 31], and discuss how these processes impact SARS‐CoV‐2 infection and COVID‐19 severity.

The immune system has evolved to respond to infection and protect the host against pathogens. The innate arm of this immune system detects components of pathogens, such as lipopolysaccharide and double‐stranded RNA to mount an early response before the adaptive arm of the immune system develops pathogen‐specific responses and long‐term memory. Studies have found that the innate immune response is compromised in older people. For instance, neutrophils exhibit impaired cytokine signaling, production of peroxide and nitric oxide, pattern recognition receptor signaling, and phagocytic function [31, 32]. Similarly, macrophages have reduced cytokine production, chemotaxis, and phagocytic functions. On the other hand, natural killer (NK) cells exhibit age‐related population shifts from a less mature, cytokine‐producing subset to a mature subset with reduced migration, cytokine secretion, and cytotoxicity [33]. The sensing of pathogen‐associated molecular patterns (PAMPs) by pattern recognition receptors (PRRs) in antigen‐presenting cells is also blunted in the elderly, compromising immune responses to viral infections [25].

Adaptive immune response is also substantially affected by immunosenescence [34]. Aging is associated with reduced T‐ and B‐cell responses against novel antigens from pathogens and vaccines. In brief, thymic involution and ‘exhaustion’ of naïve T cells by exposure to foreign antigens throughout life lead to a poor response to newly encountered antigens, as well as a shift toward an inflammatory, autoimmune T‐cell profile [31]. B‐cell numbers and receptor repertoire diversity are also reduced, leading to a decline in specific humoral responses against new extracellular pathogens [25, 31, 35, 36]. Besides immune cells, aging is also associated with a progressive decline in the immunological and mucociliary functions of the airway epithelium in the lungs, which may also jeopardize successful clearance of SARS‐CoV‐2 particles in older individuals [37]. Thus, immunosenescence leads to compromised innate and adaptive immune functions in older individuals, thereby increasing host susceptibility to viral infections, including that caused by SARS‐CoV‐2 [38, 39, 40, 41].

**Fig 2:**
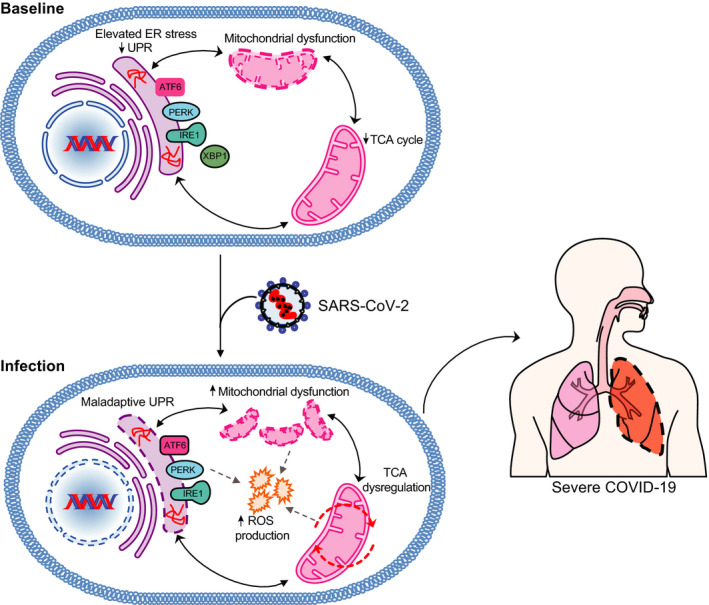
Molecular pathways underlying host tolerance to SARS‐CoV‐2. Aging results in elevated ER stress, decreased UPR capacity, mitochondrial dysfunction, and decreased TCA cycle activity that can reduce host tolerance to SARS‐CoV‐2 infection. In the context of this altered immunometabolic profile, infection with SARS‐CoV‐2 triggers a maladaptive response characterized by further mitochondrial dysfunction, maladaptive UPR, and TCA cycle dysregulation. This induces ROS production and downstream pro‐inflammatory responses that ultimately increase the risk of symptomatic outcome and severity of COVID‐19.

Following infection, the activated immune system must regulate the development of resistance with the promotion of host tolerance to restore health and avoid complications from excessive end‐organ damage. Imbalanced resistance and host tolerance could lead to exuberant systemic inflammation, especially the later stages of infection that exacerbates hypoxemia and organ dysfunction. Excessive inflammation in severe COVID‐19 is characterized by elevated levels of serum pro‐inflammatory cytokines including IL‐6, IL‐1β, IL‐2, IL‐8, IL‐17, GM‐CSF, G‐CSF, IP10, MCP1, CCL3, and TNF [3, 4, 42]. Postmortem studies of patients who succumbed to COVID‐19 have found excessive infiltration of inflammatory cells with evidence of endothelial and inflammatory cell death in multiple organs, including the lungs, heart, kidney, and intestines. These pathological findings have appeared to be particularly common in older COVID‐19 patients [43]. While the triggers and pathogenesis of excessive inflammation in COVID‐19 remain to be fully elucidated, the higher baseline levels of inflammation in older individuals may lower the tipping point for excessive inflammation following SARS‐CoV‐2 infection. This chronic low‐grade inflammation that is seen in elderly individuals is characterized by higher baseline serum concentrations of C‐reactive protein (CRP) and cytokines including IL‐6 and IL‐8. Such low‐grade inflammation may be induced by multiple factors, including impaired clearance of nondividing senescent and dead cells, chronic infections (particularly cytomegalovirus), compromised gut‐barrier functions, and obesity [23, 44, 45]. Furthermore, it has also become increasingly evident that the immune function is deeply intertwined with cellular processes governing stress response and metabolism. These interactions, an overview of which is shown in Fig. 2, are as follows:

## Inflammaging, the unfolded protein response, and oxidative stress

During viral infection, alteration of the intracellular environment provokes cellular stress responses. In particular, the endoplasmic reticulum (ER) is commandeered for synthesis of viral proteins that could overwhelm its folding capacity. Previous studies have demonstrated that CoV infection induces ER stress and upregulates expression of ER stress‐related genes including BiP, Herpud1, and GRP94 [46, 47]. In particular, the Spike (S) protein, orf3a, and orf8b of SARS‐CoV‐1 are known inducers of ER stress *in vitro* [46, 48, 49]. A recent protein interaction map also revealed that orf8 of SARS‐CoV‐2 could interact with host proteins involved in ER quality control [50], suggesting that SARS‐CoV‐2 may also manipulate the host ER machinery to facilitate viral replication in target cells. The hijacking of the host ER for viral replication and the resultant accumulation of nascent and unfolded proteins induce ER stress. Therefore, to maintain homeostasis, the unfolded protein response (UPR) is activated. Activation of the UPR by the three sensors PERK, IRE1, and ATF6 induces a downstream signaling cascade that results in production of ER chaperones, attenuation of mRNA translation, induction of ER‐associated protein degradation (ERAD), and autophagy in order to maintain folding capacity [51]. If proteostasis is not restored, prolonged ER stress eventually induces apoptosis [52]. Furthermore, inflammatory cytokines may also trigger positive feedback loops that induce ER stress in other cells to exacerbate the inflammatory response [53, 54]. As such, the activation of UPR serves to preserve tissue function and could promote disease tolerance during infection [55, 56].

The efficiency of UPR declines with age. Studies from aged mice have shown that the decline in protein folding can be attributed to increased oxidation of the molecular chaperones or reduced expression levels of UPR regulators such as GRP78 and PERK [57, 58, 59, 60]. With reduced UPR capacity in elderly individuals, ER stress triggered by viral replication would, instead of adaptive UPR, induce maladaptive UPR that is characterized by increased apoptosis and pro‐inflammatory responses. Such maladaptive UPR could thus also contribute to the immunopathogenesis of COVID‐19 [51].

Reduced UPR capacity is further compounded by aging‐associated progressive mitochondrial dysfunction [61, 62, 63]. Acting in concert with the accumulation of misfolded proteins in aged cells, mitochondrial dysfunction promotes reactive oxygen species (ROS) production and oxidative damage [64, 65]. Indeed, an integrative analysis of bulk and single‐cell transcriptomics revealed that genes associated with the mitochondrion were most negatively correlated with age [66]. Notably, some of the mitochondria‐associated genes such as NDUFB9 and NDUFAF1 were also found to interact with Orf9c of SARS‐CoV‐2 [50], suggesting that the differential expression of these mitochondria‐associated genes, along with oxidative stress in the elderly population, could potentially reduce disease tolerance and exacerbate COVID‐19 in older populations.

Although there are currently no clinical data to show that oxidative stress markers are directly correlated with COVID‐19 severity, this possibility is suggested by observations in SARS patients [67]. Expression of mitochondrial genes, stress response protein DNAJB1, and the cytokine IL‐1β were all found to be upregulated in PBMCs of SARS patients [67, 68]. In addition, *in vitro* studies suggest that ROS overproduction contributes to the pathogenesis of SARS. Specifically, SARS‐CoV 3C‐like protease has been shown to increase ROS production and apoptosis in HL‐CZ cells [69]. Moreover, the SARS‐CoV 3a protein can increase p38 MAP kinase activity and Bax oligomerization that leads to activation of the mitochondrial death pathway and apoptosis in Huh‐7 cells [70]. Collectively, these observations support that oxidative stress induced by SARS‐CoV infection contributes to disease pathogenesis. Aging‐related oxidative stress responses could thus aggravate disease severity.

The increased maladaptive UPR and oxidative stress in infected elderly patients can promote apoptosis, which can serve as a resistance mechanism to eliminate virus‐infected cells to control the spread of viruses [71]. However, apoptosis may be a double‐edged sword, as the release of damage‐associated molecular patterns (DAMPs) can further exacerbate inflammation. To limit and counteract the extent of the inflammatory response induced by cell death mechanisms, antigen‐presenting cells such as macrophages and dendritic cells are activated, which then primes the CD4+ T helper and CD8+ cytotoxic T lymphocytes to clear damaged and dying cells. In addition, macrophages can be polarized to produce anti‐inflammatory cytokines, lipid mediators, and growth factors such as interleukin‐10 (IL‐10), 15‐deoxy‐prostaglandin J_2_ (15d‐PGJ_2_), platelet‐derived growth factor (PDGF), and transforming growth factor‐β1 (TGFβ1) to promote tissue damage control and tissue regeneration [72, 73, 74]. The presence of tissue‐resident macrophages, innate lymphoid cells, and regulatory T cells can also promote tissue damage control to establish disease tolerance during infection [56]. However, both the numbers and function of the immune cells are reduced with age, resulting in an overall decline in tissue maintenance and repair capabilities that compromise disease tolerance.

Besides the increased oxidative stress levels and damage due to more ROS production, there is also diminished antioxidant capacity with age. The expression levels of antioxidant enzymes are controlled by the master transcription factor erythroid 2‐related factor 2 (NRF2) binding to the EpRE motif [75, 76]. The decline in NRF2/EpRE activity and signaling with aging can thus result in increased susceptibility to oxidative stress and damage induced by viruses including SARS‐CoV‐2 [77]. Moreover, as NRF2 is also involved in polarization of macrophage responses toward tissue damage control and mitochondrial biogenesis, the decline in NRF2 activity during aging can also compromise tissue damage control mechanisms and disease tolerance during infections [78, 79, 80]. Given the potential role of NRF2 in modulating host tolerance, NRF2 activators such as PB125® have been explored as potential therapeutics against COVID‐19 [81].

## Inflammaging and metabolic decline

Metabolism is now well established as an important regulator of susceptibility and host tolerance to viral infections. Upon SARS‐CoV‐2 infection, metabolic changes can be seen at the cellular level, where infected African Green monkey kidney cells (VeroFM) were found to have decreased tricarboxylic acid (TCA) intermediates and fructose‐6‐phosphate, with a concomitant increase in lactate production [82]. Similarly, plasma levels of malic acid and lipids such as diglycerols, free fatty acids, and triglycerols were significantly less abundant in COVID‐19 patients [83, 84]. Lactate dehydrogenase (LDH), which catalyzes the conversion of pyruvate to lactate, was also identified to be most significantly correlated with disease severity [85]. Taken together, these findings suggest that SARS‐CoV‐2 infection increases glycolytic flux and utilization of alternative sources such as free fatty acids to promote ATP production.

In response to infection, host cells are known to alter metabolic processes to protect against the damage, suggesting a plausible role for metabolism to enable disease tolerance [86]. Indeed, individuals with compromised metabolic health, including those with type 2 diabetes and other metabolic conditions, have been shown to be at greater risk of developing more severe infectious diseases [4, 6, 87, 88]; the presence of uncontrolled hyperglycemia (with or without a diagnosis of type 2 diabetes) has been found to be associated with increased mortality in COVID‐19 patients [89, 90]. In elderly individuals, metabolic processes slow down due to reduced mitochondrial performance [63]. Moreover, the loss of mitochondrial activity decreases the maximal and reserve respiratory capacity of host cells and tissues, reducing their ability to adapt to the heightened energy demand required for virus infection. Therefore, the decline in metabolic processes during aging may be maladaptive in the context of COVID‐19.

Another intriguing metabolic response to infection is that of the tryptophan–kynurenine pathway due to its immunomodulatory properties. Tryptophan is the least abundant of the essential amino acids, and its level is tightly regulated in the body. The majority of dietary tryptophan is metabolized via the kynurenine pathway, which is governed by three rate‐limiting enzymes: tryptophan 2,3‐dioxygenase (TDO), and indoleamine 2,3‐dioxygenase (IDO) 1 and IDO2 [91, 92]. TDO is constitutively expressed in the liver. In contrast, IDO1 and IDO2 are inducible enzymes expressed in nonhepatic cells such as immune cells and are upregulated by pro‐inflammatory cytokines including IFN‐γ [93, 94]. Tryptophan catabolism via the kynurenine pathway produces several metabolites that possess immunomodulatory effects. For instance, kynurenine inhibits T‐effector cell function and stimulates the production of regulatory T cells [95, 96, 97]. Moreover, other kynurenine pathway metabolites can selectively drive Th1 cells into apoptosis [98]. Indeed, these altered immune responses have been shown in mouse models of autoimmune diseases, whereby IDO deficiency or inhibition by 1‐methyl‐D‐tryptophan resulted in increased disease severity [99, 100].

Aging has been shown to be associated with increased degradation of tryptophan via the kynurenine pathway [101, 102]. Elevated levels of kynurenine have been observed in the early stages of HIV‐1 infection, which are correlated with decreased IL‐2 signaling and increased sensitivity to Fas‐mediated apoptosis in memory CD4 T cells [103]. In macaques infected with hepatitis C virus (HCV), lower levels of IDO were observed in animals that cleared the infection, whereas macaques that progressed to chronic disease showed elevated IDO levels. IDO levels in both HCV‐infected humans and macaques were positively correlated with CTLA‐4, a known inhibitory molecule of T‐cell function [104]. Likewise, serum metabolomics in a cohort of dengue‐infected patients showed that patients who progressed to severe dengue fever had increased serum kynurenine compared with individuals with mild disease or healthy controls [105]. Interestingly, a recent study on COVID‐19 demonstrated increased kynurenine levels in infected patients that were also correlated with plasma levels of the pro‐inflammatory cytokine, IL‐6 [84]. Moreover, increased kynurenine levels have been shown to elicit pain hypersensitivity and hence reduce disease tolerance in a mouse model of influenza A infection [106]. Taken collectively, these results indicate that an elevated kynurenine response during viral infection is associated with poorer clinical outcomes. As dysregulated kynurenine metabolism is observed even in healthy elderly individuals [107], this altered baseline kynurenine metabolism could predispose elderly individuals to severe disease during infection.

## Aging and physiologic changes

Fever is one of the most common symptoms of SARS‐CoV‐2 infection, and a cardinal response to many other infectious diseases [108, 109, 110]. In essence, initiation of fever occurs via activation of PRRs on innate immune cells including macrophages and dendritic cells, thereby inducing the production of host‐derived pyrogens including IL‐1β, TNF‐α, and IL‐6. These cytokines travel systemically to signal the brain’s hypothalamus to induce cyclooxygenase 2 (COX2), which in turn catalyzes the conversion of arachidonic acid into prostaglandin E2 (PGE2) that raises the thermoregulatory set point to cause fever. Previous studies in both animal models and humans have demonstrated the beneficial effects of thermoregulation for host defense against infectious diseases. Some pathogens may have reduced ability to replicate at higher body temperatures [111]. Fever leads to the sequestration of iron in host tissues, which is used by many microbes for replication [112]. Fever also helps to shape the immune response by inducing the remobilization of energy stores required to fuel microbial killing; human studies have demonstrated that metabolism is increased by ~ 13% for every 1 °C rise in temperature [113, 114, 115]. The consequence of fever is proteolysis and negative nitrogen balance in the host that results in the wasting of body tissues, to serve as a source of amino acids that are mobilized for anabolic requirements of host defense responses [110]. Also, fever induces ‘sickness behaviors’ characterized by sleepiness, fatigue, and anorexia as a means to conserve energy for the host defense responses [116]. Hence, besides reducing pathogen burden, thermoregulation also plays a critical role as a disease tolerance defense strategy.

Defects in thermoregulation or other physiologic mechanisms may thus underpin the well‐known blunted fever response in elderly patients [117, 118]. The absence of fever in acute response to viral infection could thus compromise host tolerance and hence increase the risk of severe disease outcomes [119]. Given the protective role of thermoregulatory mechanisms on infectious diseases, questions have been raised regarding the safety and risk of using antipyretics (such as nonsteroidal anti‐inflammatory drugs and paracetamol) for the management of fever symptoms in elderly COVID‐19 patients [120]. Indeed, a retrospective analysis in South Korea suggested that NSAID use is associated with increased risk of a primary composite outcome consisting of in‐hospital death, intensive care unit admission, ventilator use, and sepsis in adults hospitalized with COVID‐19 [121]. However, the roles of thermoregulation on the pathogenesis of COVID‐19 remain to be established. Clinical studies to date have not yielded sufficient evidence to determine whether the use of antipyretics for the short‐term management of fever in patients with suspected or confirmed COVID‐19 could pose additional harm [122, 123].

Another potentially important aspect of physiological changes in aging is in the renin–angiotensin pathway; this pathway contributes to essential hypertension, the prevalence of which increases with age. A key component in the regulation of this pathway is the SARS‐CoV‐2 receptor, angiotensin‐converting enzyme 2 (ACE‐2). Studies in mice found that ACE‐2 was downregulated in the lungs of mice after SARS‐CoV‐2 induced lung injury [124, 125, 126, 127], mediated at least in part through the activity of IL‐4 and IFN‐γ [128, 129, 130]. Interestingly, ACE‐2 expression has also been found in rats to be reduced with increasing age [131]. While ACE‐2 downregulation may promote host resistance to SARS‐CoV‐2 infection, there is a potential trade‐off that compromises disease tolerance. ACE‐2 is a carboxypeptidase that cleaves angiotensin I (Ang I) into Ang1‐9 and angiotensin II (Ang II) into Ang1‐7 [132, 133]. Downregulation of ACE‐2 would therefore result in increased Ang II levels in the lungs that would promote vascular permeability and severe acute lung injury due to Ang II type 1a receptor activation [124]. Indeed, this may explain why severe COVID‐19 patients seem to become negative for SARS‐CoV‐2 by RT–PCR more quickly than mild cases of COVID‐19. These observations also hint at the possibility that intervention strategies that augment ACE2 physiological functions, or the supplementation of soluble recombinant ACE2 proteins to infection sites may potentially aid in neutralizing SARS‐CoV‐2 infection and promote host tolerance to the disease [124, 134].

## Environmental factors

The availability of nutrients, including glucose, amino acids, and fatty acids, is essential for generating energy and macromolecules required for the induction of innate and adaptive immunity, as well as for cell proliferation needed for tissue repair. In addition, micronutrients (e.g., iron, zinc, and magnesium) are required for nucleic acid synthesis and antioxidant defenses (e.g., vitamin C and E) [135, 136, 137], suggesting that the levels of micronutrients may also influence host tolerance and disease outcome of viral infections. Some micronutrients such as vitamin D have been demonstrated to directly impact immune cell functions and the induction of an antiviral state [138, 139]. Nutrition can also impact the gut microbiota, which can potentially impact host resistance and tolerance to viral infections [140]. Given the role of nutrition in immune function, it is tempting to speculate that nutrition will affect host resistance and disease tolerance during SARS‐CoV‐2 infection. This consideration will be relevant particularly in aged individuals where the mobilization of body nutrient stores is less effective, and the metabolic processes are slower.

As approximately 15% of the COVID‐19 patients can die from secondary bacterial infections and the elderly are at increased risk [9], antibiotics are often prescribed for patients with severe disease manifestations. Thus, besides increasing the risk of antibiotic resistance development, antibiotics may indirectly reduce host tolerance to SARS‐CoV‐2. This is because antibiotics can induce mitochondrial damage, or inhibit mitochondrial activity and biogenesis [141, 142] that can compromise cell bioenergetics and ATP production, consequently priming infected cells to be more susceptible to cell death [143]. It is thus thought that the reduction in the use of antibiotics could be beneficial in COVID‐19 patients. In addition, therapies that promote mitochondrial activity could be a possible treatment for elderly COVID‐19 patients.

## Potential therapeutic approaches that promote host tolerance

Thus far, we have discussed that aging can lead to reduced immune functions, decreased UPR capacity, increased susceptibility to oxidative stress, and metabolic decline that can potentially compromise host tolerance to COVID‐19 (Fig. 2). These pathways are highly interconnected, and viral infections could result in further disruption of ER stress and host cell metabolism that induces pro‐inflammatory and oxidative stress responses involved in viral pathogenesis (Fig. 2). Indeed, previous studies from our laboratory support this notion. We found that individuals with lower expression levels of genes in the adaptive ER stress pathway, at baseline, were more likely to tolerate infection with a live attenuated yellow fever virus (YF17D) and thus remain asymptomatic. By contrast, those with elevated expression of genes in the ER stress pathway at baseline were more susceptible to symptomatic YF17D infection [144]. Such individuals responded to YF17D infection by inducing maladaptive UPR; the pro‐inflammatory responses were statistically correlated with symptomatic outcome. Reduced capacity to maintain proteostasis could thus also be an underpinning of COVID‐19 in the elderly.

As the decline in resistance and disease tolerance mechanisms can predispose older adults to increased risk of severe disease, the application of therapeutics that restore such homeostatic mechanisms could be useful as an adjunctive treatment for such COVID‐19 patients. In particular, drugs that either lower ER stress, improve UPR capacity, inhibit ROS and downstream oxidative stress responses, or reduce glycolytic flux could all serve to reduce the pro‐inflammatory response that underpins COVID‐19 pathogenesis. Indeed, interactome studies of SARS‐CoV‐2 have identified several drugs that can potentially target these processes, such as rapamycin, metformin, and haloperidol [50, 145]. Targeting these pathways could thus augment the anti‐inflammatory approaches that are currently explored or used, such as dexamethasone [146], to treat COVID‐19 patients and prevent the inflammation‐driven ARDS and systemic complications.

As metabolic pathways can influence symptomatic outcome of viral infections and a key energy sensor is the enzyme 5′ AMP‐activated protein kinase (AMPK) [147, 148], the activation of AMPK at baseline may thus promote disease tolerance. Under low energy states as manifested by increased AMP/ATP and ADP/ATP ratios, AMPK is activated by upstream kinases, leading to the upregulation of catabolic pathways and downregulation of anabolic pathways in order to maintain energy homeostasis [147, 149, 150]. In addition, AMPK has been shown to promote autophagy and regulate mitochondrial health [151, 152]. Activation of AMPK has also been shown to improve outcomes in several models of disease including hyperglycemia in type 2 diabetes, nonalcoholic fatty liver disease (NAFLD), diabetic nephropathy, and atherosclerosis [153, 154, 155, 156].

To capitalize on the potential of AMPK as a therapeutic target, an understanding of how AMPK function changes during aging is required. Using Fisher 344 rats, Reznick *et al*. elegantly demonstrated that upon 5′‐aminoimidazole‐4‐carboxamide‐1‐β‐D‐ribofuranoside (AICAR) stimulation, AMPK activity in older animals is reduced compared with younger animals [157]. This reduction in activity is not due to decreased expression of AMPK or activity and levels of the upstream activator liver kinase B1 (LKB1). In addition, reduced mitochondrial biogenesis and phosphorylation of the AMPK substrate acetyl‐CoA carboxylase (ACC) were noted in older rats. Likewise, using biopsies of the vastus lateralis muscle from younger (aged 19–41) and older (aged 64–86) human males, AMPK activity and phosphorylation were observed to be lower in older adults and are associated with decreased phosphorylation of ACC [158]. Interestingly, senescent primary human CD8 T cells demonstrate reduced autophagy that fails to increase upon nutrient starvation [159]. This failure in autophagy is correlated with increased apoptosis and DNA damage, supporting previous works that showed the essential role of autophagy in promoting T‐cell survival through clearance of damaged mitochondria and homeostasis of naïve T‐cells [160]. Collectively, these studies suggest that aging‐related decline in AMPK activity contributes to mitochondrial dysfunction, altered lipid metabolism, and dysfunctional autophagy and that therapeutics that promote AMPK activity at baseline could improve host tolerance to viral infections.

Another appealing metabolic target is the kynurenine pathway, which can improve host tolerance given its immunomodulatory effects. Cancer biologists have explored compounds that target the kynurenine pathway as potential immunometabolic adjuvants that act in concert with other therapeutics such as immune checkpoint inhibitors and cancer vaccines to boost antineoplastic responses. Several of these drugs have been tested in phase I and phase II clinical trials, some of which demonstrate significant therapeutic activity [161, 162]. With regard to infection, the plausibility of targeting the kynurenine pathway to modulate host responses has been illustrated in several *in vitro* and animal models. For example, primary nasal epithelial cells treated with the IDO‐1 inhibitor 1‐D‐methyl tryptophan (1‐MT) showed reduced production of pro‐inflammatory cytokines including IL‐7 and G‐CSF upon infection with the PR8 strain of influenza A virus [163]. Likewise, C57BL/6 mice treated with 1‐MT demonstrated increased influenza‐specific CD4, CD8, and effector memory T‐cell numbers in the lung parenchyma after infection with the A/HKx/31 influenza A strain [164]. Similarly, in a mouse model of HIV‐1 encephalitis, 1‐MT treatment resulted in increased levels of CD8 T cells in both peripheral blood and areas of the brain containing HIV‐1‐infected monocyte‐derived macrophages (MDMs) compared with untreated controls [165]. Furthermore, treated mice showed greater clearance of infected MDMs 3 weeks postinfection compared with untreated mice. Collectively, these studies highlight the potential of modulating the kynurenine pathway as a means to improve host tolerance. However, it should be noted that the drug used in these studies (1‐MT, i.e., indoximod) has poor bioavailability in humans [166]. Moreover, further studies have indicated that indoximod may have other mechanisms of action that contribute to its immunomodulatory effects [167]. More studies are required with specific IDO‐1 inhibitors such as epacadostat to determine whether IDO‐1 inhibition would be a suitable method for improving host tolerance.

In the elderly, the decline in Nrf2 activity can make them more susceptible to oxidative stress responses triggered by viral infections or chronic diseases [168, 169]. Therefore, Nrf2 activators could potentially enhance host tolerance to SARS‐CoV‐2 in elderly patients, as this would induce antioxidant enzymes and suppress inflammation and oxidative stress responses involved in disease pathogenesis. Indeed, exposure of human cells to the Nrf2 activator PB125^®^ *in vitro* has been shown to reduce endotoxin‐induced cytokine storm [81]. Interestingly, PB125^®^ also downregulates the ACE2 and TMPRSS2 mRNA expression, which are both involved in mediating SARS‐CoV‐2 entry in host cells. However, clinical trials will be needed to evaluate the effectiveness of Nrf2 activators to reduce the risk of severe COVID‐19.

## Conclusions and perspectives

When the next pandemic occurs, it will be unlikely that an effective vaccine or antiviral drug will be immediately available to protect us from getting infected, as the pathogen involved will be unknown. Therefore, supportive care to promote disease tolerance, especially in elderly patients who are more susceptible to infections, will remain to be an important first line of defense to improve patient health. The COVID‐19 pandemic has reinforced the notion that elderly individuals are at the highest risk of severe disease due to compromised resistance and disease tolerance arising from reduced immune functions, weakened UPR, decline in mitochondrial functions, physiological alterations, slower metabolism, and environmental perturbations. It is thus time for us to begin to understand the underlying molecular mechanisms underlying host tolerance to viral infections, to enable the development of therapies to treat infected elderly patients, which will better prepare us for the next pandemic. Some of these approaches to enhance host defense could involve boosting host immunity, UPR, mitochondrial functions, homeostatic regulation, and host metabolism. Future studies will provide insights into the molecular pathways that would be most effective in promoting host tolerance to viral infections.

## Conflicts of interest

The authors declare no conflict of interest.

## Author contributions

DZLM and KRC conceptualized the review. DZLM, CYYC, and KRC wrote the manuscript. KRC and EEO reviewed and revised the manuscript.
